# Effect of motion-graphic video-based training on the performance of operating room nurse students in cataract surgery in Iran: a randomized controlled study

**DOI:** 10.3352/jeehp.2023.20.34

**Published:** 2023-11-28

**Authors:** Behnaz Fatahi, Samira Fatahi, Sohrab Nosrati, Masood Bagheri

**Affiliations:** 1 Department of Operating Room, School of Allied Medical Sciences, Kermanshah University of Medical Sciences, Kermanshah, Iran; 2 Center for Educational Research in Medical Sciences (CERMS), Department of Medical Education, School of Medicine, Iran University of Medical Sciences, Tehran, Iran; 3 Clinical Research Development Center, Imam Khomeini and Mohammad Kermanshahi and Farabi Hospitals, Kermanshah University of Medical Sciences, Kermanshah, Iran; Hallym University, Korea

**Keywords:** Training, Medical field, Students, Operating rooms, Cataract extraction

## Abstract

**Purpose:**

The present study was conducted to determine the effect of motion-graphic video-based training on the performance of operating room nurse students in cataract surgery using phacoemulsification at Kermanshah University of Medical Sciences in Iran.

**Methods:**

This was a randomized controlled study conducted among 36 students training to become operating room nurses. The control group only received routine training, and the intervention group received motion-graphic video-based training on the scrub nurse’s performance in cataract surgery in addition to the educator’s training. The performance of the students in both groups as scrub nurses was measured through a researcher-made checklist in a pre-test and a post-test.

**Results:**

The mean scores for performance in the pre-test and post-test were 17.83 and 26.44 in the control group and 18.33 and 50.94 in the intervention group, respectively, and a significant difference was identified between the mean scores of the pre- and post-test in both groups (P=0.001). The intervention also led to a significant increase in the mean performance score in the intervention group compared to the control group (P=0.001).

**Conclusion:**

Considering the significant difference in the performance score of the intervention group compared to the control group, motion-graphic video-based training had a positive effect on the performance of operating room nurse students, and such training can be used to improve clinical training.

## Graphical abstract

[Fig f2-jeehp-20-34]

## Introduction

### Background/rationale

The operating room is considered the main clinical education setting for operating room nurse students [1], and internship provides them with the opportunity to experience their theoretical knowledge in a real setting and at the patient’s bedside [2]. Many studies have shown that most errors in the operating room occur during initial learning, the main reason for which is learners’ weak knowledge [1,3]. Inadequacy of clinical experiences during education may increase the level of anxiety and fear of being unable to do clinical work and, as a result, reduce the level of students’ learning and increase related errors [4].

Cataracts are the cause of blindness for more than a third of the 36 million blind people around the world [5]. Phacoemulsification is one of the most common and modern methods of cataract treatment and is also increasingly performed in Iran [6]. Using an improper technique for cataract surgery and failing to observe the principles of asepticity and improper preparation and draping can cause endophthalmitis [5]. Therefore, cataract surgery training is a priority for the health system [7], but students in internship for eye surgery have a limited understanding of it due to the limitations of surgery and its complex topographical anatomy. The increasing number of students, the limited duration of internships, the small number of clinical educators, and the lack of clinical cases for observation and practice make students have fewer learning opportunities [8].

Motion graphics are an emerging technology that has gained popularity due to its excellent ability to attract attention and direct the mind of the audience toward a given objective [9]. Although observational learning is the most stable and effective type of learning, the most effective teaching method involves activating the learner himself or herself in the teaching-learning process [10]. It will be useful to implement motion-graphic video training to improve students’ understanding of specific procedures.

### Objectives

The present study aimed to determine the effect of motion-graphic video training on the performance of operating room nurse students as scrub nurses in cataract surgery using phacoemulsification at Kermanshah University of Medical Sciences. The specific objective was to compare the amount of change in students’ performance status in both control and intervention groups. It was hypothesized that using motion-graphic videos as a teaching tool would improve students’ clinical skills.

## Methods

### Ethics statement

This study was approved by the Ethics Committee of Kermanshah University of Medical Sciences (Ref. ID: IR.KUMS.REC.1401.403) and the Clinical Trials of Iran (Ref. ID: IRCT20211204053270N1), and informed consent was obtained from the students to participate in this study.

### Study design

This randomized controlled study was described according to the CONSORT (Consolidated Standards of Reporting Trials) statement (available at: https://www.consort-statement.org/).

### Setting

The present study consisted of a pre-test and post-test design with a control group. This study began on February 13, 2023 and ended on June 27, 2023, at the Imam Khomeini Educational Hospital in Kermanshah.

### Interventions

The students in both groups received the necessary explanations about cataract surgery using phacoemulsification from the relevant educator in a routine manner at the beginning. In the control group, the students’ practical skills as the first scrub nurse were recorded through a researcher-made checklist in the form of a pre-test on the first day and a post-test on the last day of the internship (interval of 8 days) by an evaluator outside the research team who was not aware of the division of students into groups. The control group only received routine training from the educator at the beginning, and a motion-graphic video was not played for them. On the first day of the internship, the students in the intervention group applied their knowledge as first scrub nurses in cataract surgery using phacoemulsification, and their practical skills were recorded by the checklist in the form of a pre-test by the evaluator, who was the same person at all stages of the research. The checklist was completed by the evaluator through a mobile phone, and the students were also unaware of the evaluator’s presence in the room and his evaluation of their performance. Then on the first day, a motion-graphic video was announced by the educator (researcher) to the students of the intervention group and then made available to them, who watched it as many times as desired. Then, on the last day of the internship (8 days after the intervention), the students went to work as scrub nurses, and their performance was recorded in the post-test through the checklist. A 12-minute motion-graphic video (combination of filming and graphics) of cataract surgery using phacoemulsification was prepared (Supplement 1). Its content was based on references for the operating room field under the supervision of an experienced ophthalmic surgeon and included identifying surgical tools, setting up the surgical table, preparing surgical drugs, preparation and draping, handing over the tools to the surgeon during the surgical process, and so forth.

It should be noted that the students of the control group were first included in the study to prevent interference and sharing of educational content between the students of the 2 groups, and the students in each internship group in the intervention group were also told not to talk about the educational video with other students in the same course. To adhere to the principles of educational justice, at the end of the study, the designed motion-graphic video was also provided to the students of the control group.

### Participants

The statistical population included all students in the sixth and eighth semesters at Kermanshah University of Medical Sciences in the academic year 2022–2023. The inclusion criteria included passing the theoretical unit on eye surgery technology. The exclusion criteria included unwillingness to continue cooperation and having clinical work experience in the field of eye surgery.

### Outcomes

In this study, the following outcomes were investigated: demographic characteristics and the intervention and control groups’ scores for performance as operating room nurse students in cataract surgery using phacoemulsification with a checklist.

### Data sources/measurement

To measure the performance of students in cataract surgery using phacoemulsification, a researcher-made checklist of evaluations of the performance of scrub nurses in cataract surgery using phacoemulsification was used (Supplement 2). This checklist included 30 criteria scored on a 3-point scale, with 0 indicating failure to perform, 1 indicating incomplete performance, and 2 indicating correct performance. This checklist examined the performance of the scrub nurses in cataract surgery using phacoemulsification before, during, and after surgery. The content of the checklist was given to 10 faculty members of the operating room department of Kermanshah University of Medical Sciences, and after making the necessary corrections, its validity (content validity index=1, content validity ratio=0.95) was confirmed and its reliability was confirmed by a Cronbach α of 0.95.

### Bias

None.

### Study size

Sample size estimation was not done since all target students were included. In a post-hoc analysis using G*Power 3.1.9.7 (http://www.gpower.hhu.de/), the power (1–β probability) was calculated as 1.0 using the following input parameters: 2-tailed t-test; effect size (D), 4.6; α error probability, 0.05; and a sample size of 18 for both groups 1 and 2.

### Randomization

The students were randomly divided into 2 intervention and control groups by the block method and a computerized list of random numbers. To allocate the participants to the research groups, a computerized list of random numbers was specified by a statistical consultant to randomly allocate the subjects in pairs.

### Blinding (masking)

No blinding was done.

### Statistical methods

The obtained information was analyzed using IBM SPSS ver. 25.0 (IBM Corp.), and the significance level was considered 0.05. To describe the demographic information of the subjects and research variables, descriptive statistics such as mean, standard deviation, and percentage of frequency, and graphs were used, as well as the t-test and chi-square test. Then, the Shapiro-Wilks (or Kolmogorov-Smirnov) statistical test was used to assess the normality of the data distribution, and the Levene test was used to evaluate the homogeneity of variance in the groups. Analysis of covariance was conducted to compare the performance of students in both groups before and after the intervention.

## Results

### Participants

Women comprised 48.3% of participants in the control group and 51.7% of participants in the intervention group. The mean age in the control group was 22.56 years and 22.78 years in the intervention group. The chi-square test showed no significant difference between the groups in the distribution of gender (P=0.674), marital status (P=0.070), and academic semester (P=1.00). Therefore, the groups were homogeneous regarding the distribution of variables of gender, marital status, and academic semester. Also, the independent t-test showed that the mean age (P=0.802) and the grade point average (GPA) (P=0.798) did not differ significantly between the groups, meaning that the groups were homogeneous for the average age and GPA (Table 1).

### Main results

According to the results of the paired t-test, the mean difference between the pre-test and post-test in the control group was 8.61 (P=0.001) and 32.61 (P=0.001) in the intervention group (Table 2).

According to the results of the dependent t-test, the mean difference between the pre-test scores of the control and intervention group was 0.214 (P=0.832) and the mean difference between the post-test of the control and intervention groups was 13.737 (P=0.001) (Table 3).

**Fig. 1.**
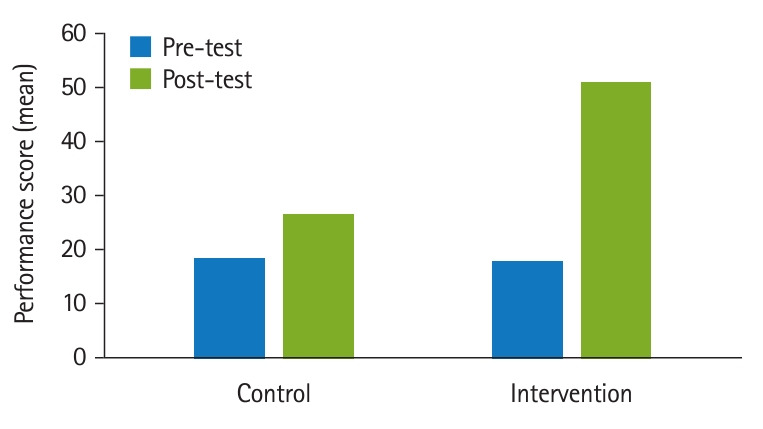
Comparison of pre-test and post-test performance scores of operating room nurse students in cataract surgery in the control and intervention groups

The results of the present study showed that the pre-test mean performance of operating room nurse students in cataract surgery showed no significant difference between the control and intervention groups, and the line indicates stability. Meanwhile, the post-test mean performance of operating room nurse students in cataract surgery showed a significant difference between the control and intervention groups, with a greater improvement in the intervention group than in the control group (Fig. 1).

Data are available at Dataset 1.

## Discussion

### Key results

The result of this study shows that the motion-graphic video-based training had a significant positive effect on the performance of operating room students, and the use of motion-graphic video-based training as a teaching tool has helped operating room students in cataract surgery better understand the content and improve their abilities and skills.

### Interpretation

The results of this study showed that the intervention (motion-graphic video-based training) had a significant positive effect on the performance of operating room nurse students, and the use of motion-graphic video-based training as a teaching tool helped operating room nurse students in cataract surgery better understand the content and improve their abilities and skills.

According to the results of the present study, the post-test mean performance of operating room nurse students in cataract surgery showed a significant difference between the control and intervention groups. Therefore, in clinical training, we can hire educators and use motion-graphic videos to improve. The results of the present study showed that the difference between the pre-test and post-test mean performance of operating room nurse students in cataract surgery was significant in the control group. The importance of this result is that it shows that factors other than motion-graphic video-based training may have an effect on improving the performance of the control group. One of these factors is the time effect, according to which the passage of time itself can change performance. In other words, it is possible that the performance of the control group improved simply over time, unrelated to the study intervention. Another factor is the learning effect. The control group may have experienced a learning effect over time. Even without receiving a specific intervention, repeated exposure to the same task (surgery) can lead to improved performance through learning and familiarity with surgery-related issues.

### Comparison with previous studies

In this regard, a quasi-experimental study conducted by Mirmoghtadaie et al. [11] in 2019 among pharmacology students of Tehran University of Medical Sciences showed a significant difference between the mean scores of the experimental and control groups in the post-test, and the use of animations had a positive effect on the knowledge and attitude of pharmacology students.

### Limitations

The limitations of this search include the sensitivity of the eye surgery field and the use of operating room nurse students in 2 different 2 semesters.

### Generalizability

The results of the present study can be useful for clinical educators to better train operating room nurse students in other fields of surgery and other students of medical sciences in hospitals.

### Suggestions

Empowering operating room nurse students as an efficient future workforce is very important for the reduction of errors in the operating room. Therefore, future studies are recommended to investigate the effectiveness of motion-graphic video-based training in other fields of surgery and compare this method with other methods of training.

### Conclusion

Motion-graphic video-based training significantly affected the performance of operating room nurse students in cataract surgery using phacoemulsification. Compared to the control group, a significant difference was found in the mean performance of students in the intervention group. These results showed that the educational intervention had a positive and significant effect on improving students’ performance.

## Figures and Tables

**Figure f2-jeehp-20-34:**
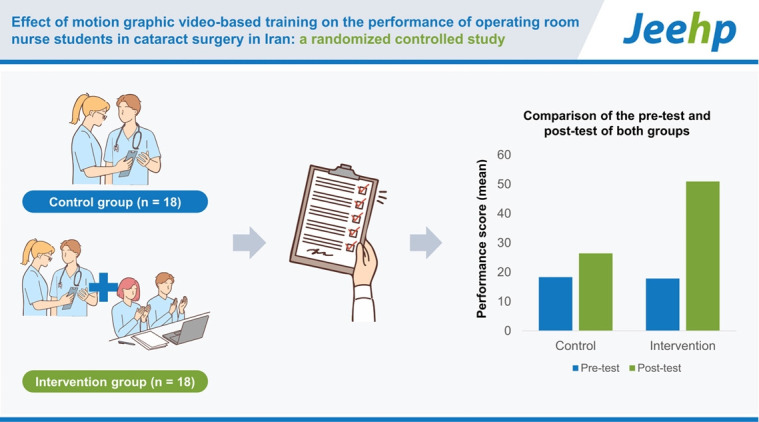


**Table 1.** Demographic characteristics of the research samples in the control and intervention groups

Characteristic	Total	Group		P-value
		Intervention	Control	
Gender				0.674
Female	29 (80.6)	15 (51.7)	14 (48.3)	
Male	7 (19.4)	3 (26.7)	4 (22.2)	
Total	36 (100.0)	18 (50.0)	18 (50.0)	
Marital status				0.070
Single	33 (91.7)	18 (16.7)	15 (45.5)	
Married	3 (80.6)	0	3 (16.7)	
Total	36 (100.0)	18 (50.0)	18 (50.0)	
Semester				1.00
6	16 (44.4)	8 (50.0)	8 (50.0)	
8	20 (56.4)	10 (50.0)	10 (50.0)	
Total	36 (100.0)	18 (50.0)	18 (50.0)	
Age (yr)		22.78±2.81	22.56±2.45	0.802
Grade point average		16.93±1.18	17.03±1.07	0.798

Values are presented as number (%) or mean±standard deviation.

**Table 2.** Intra-group comparison of pre- and post-test performance scores of operating room nurse students in cataract surgery between the control and intervention groups

Group	Mean±SD	Minimum	Maximum	t-value	P-value	95% CI of the difference
Control (n=16)				-8.841	0.001	-10.66 to -6.55
Pre-test	17.83±6.81	8	30			
Post-test	26.44±6.37	18	38			
Intervention (n=16)				-32.077	0.001	-34.75 to -30.46
Pre-test	18.33±7.21	8	31			
Post-test	50.94±4.07	42	58			

SD, standard deviation; CI, confidence interval.

**Table 3.** Intergroup comparison of pre- and post-test performance scores of operating room nurse students in cataract surgery between the control and intervention groups

Phase	Mean±SD	Minimum	Maximum	t-value	P-value	95% CI of the difference
Pre-test				-0.214	0.832	-5.25 to 4.25
Control	17.83±6.81	8	30			
Intervention	18.33±7.21	8	31			
Post-test				-13.737	0.001	-28.12 to -20.87
Control	26.44±6.37	18	38			
Intervention	50.94±4.07	42	58			

SD, standard deviation; CI, confidence interval.

## Footnotes

**Data availability**
Data files are available from Harvard Dataverse: https://doi.org/10.7910/DVN/RVOPVT Dataset 1. Raw data of participants for each item of the pre- and post-tests.

## Supplementary materials

Supplementary files are available from Harvard Dataverse: https://doi.org/10.7910/DVN/RVOPVT

Supplement 1. Motion-graphic video used for training nursing students’ to perform as scrub nurses in cataract surgery using phacoemulsification.

Supplement 2. A check list for evaluating the performance of students as scrub nurses in cataract surgery using phacoemulsification.

Supplement 3. Audio recording of the abstract.
