# Supplementary material for: Effect of motion-graphic video-based training on the performance of operating room nurse students in cataract surgery in Iran: a randomized controlled study
Source: J Educ Eval Health Prof. 2023 Nov 28;20:34. doi: 10.3352/jeehp.2023.20.34 (PMC11009010; doi:10.3352/jeehp.2023.20.34)
Supplement: Supplementary file 3 — Supplement 2. A check list for evaluating the performance of students as scrub nurses in cataract surgery using phacoemulsification. [file jeehp-20-34-suppl2.docx]

**Supplement 2.** The checklist of evaluation of performance of scrub nurse in cataract surgery using phacoemulsification

**Demographic information**

**• Age:**

**• Sex: Male □ Female □**

**• Grade point average:**

**• Semester:**

| **Row** | **Item to be evaluated** | **It does not** | **It does imperfectly** | **It does it right** |
| --- | --- | --- | --- | --- |
|  |  | **0** | **1** | **2** |
| 1 | Wearing a surgical gown |  |  |  |
| 2 | Wearing gloves in a closed manner |  |  |  |
| 3 | Preparation of hydro dissection solution |  |  |  |
| 4 | Preparation of required drugs |  |  |  |
| 5 | Preparation of surgical site |  |  |  |
| 6 | Draping of surgical site |  |  |  |
| 7 | Paste the eye perforation |  |  |  |
| 8 | Placing an eye speculum to move the eyelid. |  |  |  |
| 9 | Information about the position of the surgical table and scrub person |  |  |  |
| 10 | Arrange the surgical table based on priority |  |  |  |
| 11 | Assembling the components of the phaco pen |  |  |  |
| 12 | Delivery of the knife (3.2 mm) |  |  |  |
| 13 | Injection delivery into the anterior chamber |  |  |  |
| 14 | Delivery of trypan blue injection to paint the anterior capsule |  |  |  |
| 15 | Timely washing of the eyes |  |  |  |
| 16 | Delivery of rhexis forceps for capsulorhexis |  |  |  |
| 17 | Delivery of the knife and colibri forceps for the main incision |  |  |  |
| 18 | Delivery of hydro dissection solution to separate the lens from the posterior capsule |  |  |  |
| 19 | Delivery of phaco pen and chopper to crush the cloudy lens |  |  |  |
| 20 | Assembling the double cannula and delivering it to harvest the lens cortex |  |  |  |
| 21 | Eye gel delivery |  |  |  |
| 22 | Assembling and delivering of lens, injector, and cartilage |  |  |  |
| 23 | Delivery of secondary or sinskey hook for maneuvering the secondary lens |  |  |  |
| 24 | Delivery of double cannula to clean the inside of the eye |  |  |  |
| 25 | Delivery of acetylcholine to close the pupil of the eye |  |  |  |
| 26 | Delivery of stroma hydration solution to close the incision |  |  |  |
| 27 | Delivery of sub conjunctival injection |  |  |  |
| 28 | Dressing the operating site |  |  |  |
| 29 | Separation the components of the phaco pen |  |  |  |
| 30 | Wash the used surgical instruments |  |  |  |
